# Excessive smartphone use increases self-reported auditory and vestibular symptoms

**DOI:** 10.1007/s00508-024-02418-1

**Published:** 2024-08-23

**Authors:** Emre Söylemez, Mehmet Dağ, Abdulkadir Ilgaz, Bekir Korkmaz, Ümit Topçuoğlu, Ahmet Düha Koç, Serdar Ensari

**Affiliations:** 1 Faculty of Medicine, Department of Otorhinolaryngology, Karabuk University, Karabuk, Türkiye; 2 Vocational School of Health Services, Karabuk University, Karabuk, Türkiye; 3 Faculty of Health Sciences, Karabuk University, Karabuk, Türkiye; 4 Faculty of Medicine, Department of Otorhinolaryngology, Ankara Yıldırım Beyazit University, Karabuk, Türkiye

**Keywords:** Hearing, Tinnitus, Balance, Anxiety, Smartphone addiction

## Abstract

**Background:**

With widespread smartphone use, there is growing concern about their potential impact on human health.

**Objective:**

The effects of smartphone use on self-reported hearing ability, tinnitus, balance, falls, and anxiety level were investigated in this study.

**Methods:**

This study included 682 participants who were divided into 2 groups: a high smartphone use (HSU) group and a low smartphone use (LSU) group. Both groups were evaluated for hearing ability using the Amsterdam inventory for auditory disability and handicap; balance status using the vertigo, dizziness, imbalance symptom scale; anxiety status using the Beck anxiety index; and fall and tinnitus status using the visual analog scale.

**Results:**

The HSU group showed significantly worse hearing ability, tinnitus, balance, falling, and anxiety status results than the LSU group (*p* < 0.001). There was a positive correlation between smartphone addiction severity and auditory impairment, tinnitus, risk of falling, and anxiety, as well as a negative correlation with balance score (*p* < 0.001).

**Conclusion:**

The findings suggest that individuals with excessive smartphone use are more likely to experience hearing, tinnitus, balance, falling, and anxiety problems than those who use smartphones less frequently. Excessive smartphone use may be considered a potential risk factor for these problems.

## Introduction

Smartphones are undoubtedly one of the greatest inventions of recent times. These devices enable us to do much more than communicate; we can play games, listen to music, surf the internet, and perform many other activities [1]. Smartphones make our lives easier and enhance our quality of life. Their ease of use and accessibility make them appealing to people of all ages. In Germany, 93% of children and young people between the ages of 12 and 19 years own a smartphone, and 92% of people use their devices daily, with an average daily usage time of approximately 3.5 h [2]. The rapid increase in smartphone usage and the multitude of features they offer have highlighted the issue of smartphone addiction [3]. Despite the lack of official diagnostic criteria for smartphone addiction, it is considered a form of behavioral addiction. It is similar to, but more common than, internet addiction [4].

Excessive smartphone use can lead to physical health problems such as blurred vision and pain in the neck or wrists [5]. Additionally, excessive smartphone use can cause various mental and behavioral problems, including maladaptive behavior, decreased performance in school or work, reduced real-life social interactions, and relationship disorders [6]. A study stated that excessive phone use increases the level of depression and anxiety and causes poor sleep quality [1].

There is increasing public concern about the possible adverse health effects of mobile radiation exposure, which seems justified given the prevalence of smartphone use [7, 8]. Unlike ionizing radiation, mobile phone radiofrequency (RF) does not have enough energy to break chemical bonds or damage DNA [7]; however, the close proximity of a mobile handset antenna to the user’s head leads to substantial exposure to electromagnetic radiation (EMR), with approximately 40–55% of the phone’s RF energy absorbed by the user’s head [9]. Some initial studies suggested that mobile phones may increase the risk of acoustic neuroma [10, 11]. Later extensive epidemiological studies reported that mobile phones did not pose a risk of acoustic neuroma in the short term but may have some effect in the long term (>10 years) [12]. Unlike traditional mobile phones, smartphones are now used not only for making phone calls but also for messaging, video calling, playing games, following the current agenda and browsing social media. Therefore, although smartphones are often used away from the user’s head, they are used longer than traditional mobile phones. Also, smartphone features such as Wi-Fi and Bluetooth can increase the RF field.

Maintaining balance requires integrating inputs from the visual, vestibular, and proprioceptive systems into the central nervous system. High smartphone use can impact the visual system, and the sensory receptors of the audiovestibular system located in the inner ear can be influenced by both psychological factors, such as smartphone addiction, and physical factors, such as EMR exposure; however, there is limited literature on the effects of excessive smartphone use on the hearing ability and balance system, and the full extent of these effects is unclear [13–16].

This study investigated the effects of excessive smartphone use on self-reported hearing ability, tinnitus, balance, falls, and anxiety level. We hypothesize that individuals with high smartphone use will report more significant auditory impairment, tinnitus, balance problems, falls, and anxiety than those who use smartphones less frequently.

## Material and methods

### Participants

This study involved 704 university students who were asked to complete a demographic information form and disclose any additional medical conditions. Students who reported any health problems were interviewed, and their medical conditions were examined in detail. Students with true vertigo (such as benign paroxysmal positional vertigo, Meniere’s disease), neurological (except migraine), psychiatric (such as schizophrenia, major depressive disorder and bipolar disorder), systemic, orthopedic, cardiological and otological diseases (such as objective tinnitus, eardrum perforation, otalgia and hearing loss) were not included in the study. The inclusion criteria for the study were as follows: being between 18 and 50 years old, being healthy or reporting nonspecific symptoms such as subjective tinnitus and imbalance. Of the students 22 were excluded from the study because 2 had neurological disorders (epilepsy), 15 had systemic diseases (13 with diabetes mellitus and 2 with hypertension), and 5 had cardiological diseases. As a result, 682 students were included in the study. All the participants provided verbal and written consent, and the study received approval from the university ethics committee (Meeting no: 24.02.2023/02, decision no: 29).

### Procedure

To measure smartphone addiction severity, the smartphone addiction scale (SAS) was administered to all participants. Demirci et al. [17] validated and made the SAS reliable for use in Turkish, and it consists of 33 questions, each scored on a 6-point Likert scale ranging from 1 to 6. The total possible score is 196, with a high score indicating greater smartphone addiction. The participants were divided into two groups based on their SAS scores: low smartphone use (LSU) and high smartphone use (HSU) [1].

### Measurements

The participants’ balance status was assessed using the vertigo dizziness imbalance symptom scale (VDI-SS), for which Yanık et al. conducted a Turkish validity and reliability study [17]. The VDI-SS consists of items answered on a Likert scale ranging from 0 to 5, where 0 represents “all of the time” and 5 represents “none of the time.” The Cronbach’s alpha of the VDI-SS was 0.85. Participants were asked to choose the answer that best reflected their condition. A high score indicates good balance.

The frequency of falls among participants was evaluated using the visual analog scale I (VAS-I). The participants were asked to rate their frequency of falls in the past year on a scale of 0 (never fell) to 10 (fell excessively), and this rating was recorded as the VAS‑I score.

The hearing ability of the participants was assessed using the Turkish version of the Amsterdam inventory for auditory disability and handicap (T-AIADH), which contains a total of 30 questions [18]. Each question can be scored using 1 of 4 options: almost never, occasionally, frequently, and almost always. The total score ranges from 0 to 90, with higher scores indicating greater auditory disability.

The severity of tinnitus among the participants was assessed using the visual analog scale II (VAS-II). Participants were asked to rate the presence of persistent tinnitus on a scale of 0 (no tinnitus) to 10 (severe tinnitus), and this rating was recorded as the VAS-II score.

The anxiety levels of the participants were assessed using the Beck anxiety inventory (BAI), which comprises 21 questions. Each question is rated on a scale of 0 (not at all) to 3 (severely), with a total score ranging from 0 to 63.

### Statistical analysis

Statistical analysis was performed using The International Business Machines Statistical Package for the Social Science 21 (IBM SPSS Corp.; Armonk, NY, USA) software. The normality distribution of the data was assessed using the Shapiro-Wilk test. Normally distributed data were presented as mean ± SD, while non-normally distributed data were presented as median (minimum-maximum) values. The χ2 test was used to compare genders. As the data were not normally distributed, the Mann-Whitney U test was employed to compare numerical variables between groups. The Spearman correlation test was used to assess the relationship between the SAS and other variables. A significance level of 0.05 was considered statistically significant.

## Results

The mean SAS score of 682 participants included in the study was 95.78 ± 23.34 (35–175), and the median value of SAS was 94. According to the median value, the students were divided into two groups; the LHU group (SAS score < 94) and the HSU group (SAS score ≥ 94). Accordingly, 332 (37.6%) participants were included in the LHU group, and 350 (62.4%) participants were included in the HSU group. There was no difference between the groups in terms of age and gender (*p* > 0.05). The characteristics of the groups are presented in Table 1.

**Table 1:** General characteristics of the groups

	*LSU group* *n*:332	*HLU group* *n*:350	*p*
	mean ± SD or	mean ± SD or	
	median (min-max)	median (min-max)	
*SAS*	76.69 ± 12.12	113 ± 15.76	–
	79 (35–93)	111 (94–175)	
*Age (years)*	20.85 ± 3.29	20.59 ± 2.60	0.653^a^
	20 (18–47)	20 (18–45)	
*Smartphone usage time (years)*	5 (1–14)	7 (3–14)	< 0.001^a^
Gender (*n*)			0.083^b^
*Female*	244 (73.5%)	277 (79.1%)	^–^
*Male*	88 (26.5%)	73 (20.9%)	^–^
*Daily usage time (h)*	5 (1–10)	7 (3–15)	< 0.001^a^
*Purposes of use (mostly)*			
Phone calls	88 (26.5%)	94 (26.9%)	0.917^b^
Social media	218 (65.7%)	263 (75.1%)	0.007^b^
Messaging	264 (79.5%)	282 (80.6%)	0.731^b^
Gaming	85 (25.6%)	131 (37.4%)	0.001^b^
Education	26 (7.8%)	26 (7.4%)	0.843^b^
Photography and video	80 (24.1%)	92 (26.3%)	0.511^b^
*Time of smartphone use (mostly)*			
Morning	23 (6.9%)	20 (5.7%)	0.515^b^
Afternoon	47 (14.2%)	53 (15.1%)	0.716^b^
Evening	242 (72.9%)	275 (79.0%)	0.061^b^
Night	75 (22.6%)	125 (35.7%)	< 0.001^b^

*LSU* low smartphone use, *HSU* high smartphone use, *SAS* smartphone addiction scale, *a* Mann Whitney‑U test, *b* χ^2^

**Fig. 1:**
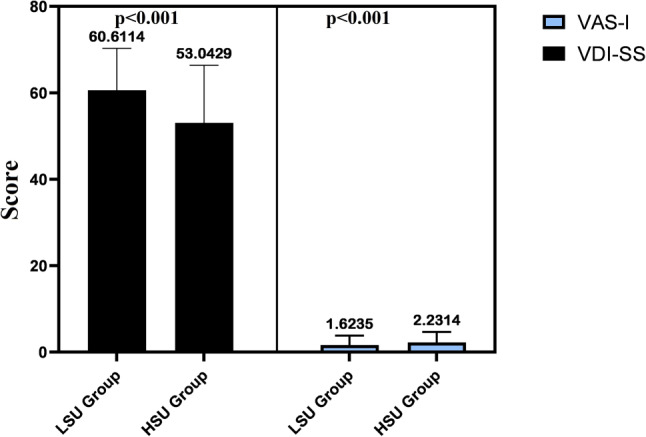
Balance and falling status according to groups. *LSU* low smartphone use, *HSU* high smartphone use, *VAS* Visual Analogue Scale, *VDI-SS* The Vertigo, Dizziness Imbalance Symptom Scale

The VDI-SS scores and falling status of individuals in the HLU group were worse than those in the LSU group (*p* < 0.05). The VDI-SS and falling states between groups are presented in Fig. 1. There was a strong negative relationship between BAI and VDI-SS (*p* < 0.001, r = −0.65). While there was a negative correlation between SAS and VDI-SS, there was a positive correlation with falling status (*p* < 0.05, Table 2).

**Fig. 2:**
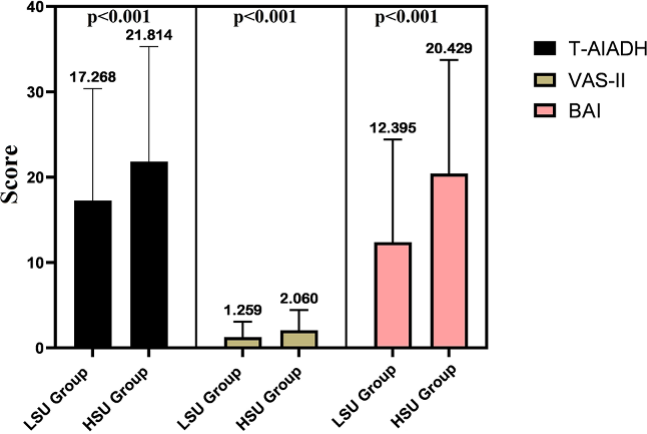
Hearing, balance and anxiety level by groups. *LSU* low smartphone use, *HSU* high smartphone use, *VAS* Visual Analogue Scale, *BAI* Beck anxiety inventory, *T‑AIADH* Turkish version of the Amsterdam inventory for auditory disability and handicap

Hearing ability, tinnitus and anxiety states of the individuals in the HLU group were worse than those in the LSU group (*p* < 0.05). The T‑AIADH, tinnitus and anxiety states between the groups are presented in Fig. 2. There was a positive correlation between the severity of smartphone addiction and T‑AIADH, tinnitus and anxiety (*p* < 0.05, Table 2).

**Table 2:** The relationship between SAS and hearing, tinnitus, falling, balance and anxiety

		SAS	
	Mean ± sd	Correlation coefficient (r)	p^*^
*VDI-SS*	56.72 ± 12.28	−0.330	< 0.001
*VAS‑I (tinnitus)*	1.93 ± 2.33	0.155	< 0.001
*T‑AIADH*	19.60 ± 13.48	0.198	< 0.001
*VAS-II (falls)*	1.67 ± 2.16	0.176	< 0.001
*BAI*	16.52 ± 13.30	0.340	< 0.001

*SAS* smartphone addiction scale, *VDI-SS* vertigo, dizziness imbalance symptom scale, *VAS‑I* visual analogue scale‑I, *T‑AIADH* Turkish version of the Amsterdam inventory for auditory disability and handicap, *VAS-II* visual analogue scale-II, *BAI* Beck anxiety inventory, ^*^Spearman correlation test

## Discussion

The increasing popularity of smartphones has raised concerns regarding their potential impact on human health. This study aimed to investigate the effects of high smartphone use on hearing ability, tinnitus, balance, falls, and anxiety. Participants in the HSU group showed worse hearing ability, tinnitus, balance, falls, and anxiety than those in the LSU group. Additionally, a positive correlation was found between the severity of smartphone addiction and auditory impairment, tinnitus, fall risk, and anxiety, while a negative correlation was observed with balance scores.

The potential effects of long-term exposure to EMR have raised significant concerns worldwide. Oktay et al. [14] conducted a study with 60 men equally divided into 3 groups (group I: those who used mobile phones for about 2 h a day for 4 years, group II: those who used mobile phones for 10–20 min a day for 4 years and group III: those who never used a mobile phone). Auditory-evoked brainstem response (ABR) and pure tone audiometry testing were applied to all participants. The study found no difference between the groups in terms of ABR results; however, the pure tone thresholds of individuals in group I (HSU) were worse than those in groups II and III. Das et al. [13] compared the hearing thresholds of the exposed and unexposed ears in individuals using mobile phones. The air and bone conduction hearing thresholds of the exposed ear were worse than those of the unexposed ear. The authors suggest that in order to establish a connection between the observed changes and the molecular and biochemical effects of EMR on the organ of Corti, further investigation into the structure of the cochlea and hair cells after prolonged exposure to mobile phones is necessary. Therefore, they recommended that additional studies be conducted. Another study [19] found that tinnitus was more common in individuals who had used mobile phones for 4 years or more, suggesting that mobile phone use could be considered a risk factor for tinnitus in future studies. In our study, all participants reported having normal hearing, and we assessed hearing ability using T‑AIADH and tinnitus using VAS. The HSU group had worse hearing ability and tinnitus than the LSU group. We chose T‑AIADH as our hearing ability assessment tool because it evaluates all five hearing functions defined by the World Health Organization (WHO): sound detection, sound discrimination, sound localization, sound lateralization, and speech discrimination [20]. Therefore, our study shows that HSU negatively affects central auditory system abilities.

Lee et al. [15] provided evidence of the potential negative impact of smartphone use on balance. The researchers evaluated the balance skills of individuals before and after playing smartphone games for 10 and 20 min using a force plate and a simulator sickness questionnaire. The study found that playing smartphone games worsened balance, but there was no significant difference between the 10-min and 20-min play time groups. The authors suggested that individuals should rest adequately after playing on a smartphone, even for just 10 min, instead of taking immediate action. Wah et al. [16] investigated the prevalence of static balance disorder and associated risk factors in smartphone users with subclinical neck pain. The authors reported that the prevalence of static balance disorder in these individuals was 74.07%. They identified 4 h of daily smartphone use, 4 years of smartphone use, and neck disability index score ≥ 7 as risk factors for static balance disorder. Our study evaluated balance using the VDI-SS and fall risk using the VAS. We found that participants with HSU had greater imbalance and fall risk. Excessive smartphone use can cause visual fatigue in individuals [21], leading to changes in the inputs from the visual system that contributes to balance. While the vestibular and proprioceptive systems can compensate for abnormal visual inputs, excessive phone use can also affect proprioceptive inputs from the cervical region due to long-term neck flexion [16]. This means that the vestibular system alone cannot compensate for abnormal visual and proprioceptive inputs.

Auditory and vestibular end organs, located in the same inner ear region and connected to each other, show anatomical and phylogenetic similarities [22]. Therefore, pathologies affecting one system can affect the other [22]. Considering the potential of EMR to affect the auditory system, the vestibular system can be affected by EMR. In studies investigating the acute effects of EMR on the vestibular system, there is no evidence of the existence of these effects [23, 24]. Pau et al. applied pulsed or continuous microwaves to individuals without vestibular complaints and evaluated the vestibular systems of these individuals with video nystagmography [23]. Small amplitude nystagmus occurred in five subjects during continuous microwave exposure and four during pulsed microwave exposure; however, the authors considered these findings insignificant. Similarly, another study reported that EMR occurring during mobile phone use did not affect the vestibular system [24]. We have not found any studies investigating the long-term effects of EMRs on the vestibular system. Knowing the long-term impact of EMR on the vestibular system can provide important information about the mechanism of EMR impact on the inner ear. On the other hand, slowly developing vestibular pathologies are compensated by the central nervous system, and individuals may not report any symptoms [25]. Therefore, the fact that the individuals with HSU in our study had more complaints of imbalance seems to be due to proprioceptive or visual interference, not vestibular interference.

Individuals’ processing capacity is limited. For this reason, during dual tasking, cognitive capacity is shared among tasks, and the error rate of individuals increases [26]. This also applies to motor or cognitive tasks performed simultaneously with smartphone use [27]. Hyong et al. [27] evaluated individuals’ balance skills during phone use (dual task) and stated that balance worsened during dual task compared to single task. The authors noted that performing another task simultaneously with a smartphone may cause falls and injuries. Moreover, individuals with HSU tend to use smartphones more while walking [28]. The insufficient resource hypothesis may explain why individuals with HSU, who already had more self-reported balance complaints than individuals with LSU in our study, fell more. Therefore, limiting smartphone use is important to prevent falls and complications resulting from falls.

Smartphones can be beneficial for relieving stress and improving mental health when used in moderation; however, excessive use can have negative effects. Demirci et al. [1] studied 319 university students and found that those who used smartphones excessively had worse anxiety, depression, and sleep quality. The authors stated that excessive smartphone use may lead to depression and/or anxiety, leading to sleep problems. Our study also found higher levels of anxiety in the HSU group, similar to the findings of Demirci et al. Smartphone ownership is associated with later bedtimes [29]. Similarly, our study showed that individuals with HSU were likelier to use smartphones at night than individuals with LSU. One study reported that exposure to mobile phone EMR in the evening may affect the pineal gland and melatonin rhythm, causing changes in cerebral blood flow and brain electrical activity [30]. Sleep is an important biological mechanism in regulating mood, and sleep disorders due to HSU can increase the level of anxiety in individuals. Additionally, individuals with psychological problems may use smartphones more to distract themselves from their mental and emotional difficulties and avoid their worries and stressors.

It is known that functional and psychiatric disorders can cause vestibular symptoms [31]. This syndrome, called phobic postural vertigo (PPV) or persistent postural-perceptual dizziness, is thought to result from the abnormal interaction between central vestibular pathways and central structures responsible for anxiety and depression. According to accepted hypotheses, balance and anxiety connections may arise from at least six neural components [32]. These neural components include afferent interoceptive information processing, a vestibulo–parabrachial nucleus network, a cerebral cortical network, a raphe nuclear-vestibular network, a coeruleo-vestibular network and a raphe-locus coeruleus loop. Abnormal interaction between these neural structures is thought to result in individuals’ greater reliance on visual or somatosensory input, increased attention to head and body movements, and more careful ambulation [33]. According to the hypothesis, individuals with PPV use these strategies, which are typically used only in high postural risk situations, in their daily routines and cannot return to normal postural strategy [31]. In our study, there was a strong negative relationship between BAI and VDI-SS, i.e., individuals with higher anxiety scores had worse balance skills (VDI-SS). This suggests that anxiety may play a mediating role in the relationship between excessive smartphone use and imbalance.

Smartphone addiction and fear of missing out (FoMO) are closely related [34]. The FoMO can be defined as feeling the need to constantly be online and check social media for fear of missing out on opportunities, experiences or events experienced by others. The FoMO can lead to problems such as anxiety, sleep disturbance, and lack of concentration [35, 36]. In our study, individuals with HSU used social media more than individuals with LSU. The FoMO can lead to individuals self-reporting hearing and balance problems, similar to HSU; however, to our knowledge, there is no research on this subject in the literature. In future studies, the effect of FoMO on self-reported hearing and balance problems, in addition to smartphone addiction, can be investigated.

This study has some limitations. The fact that the participants in our study were students and their average age was low limited our generalization of the findings. In future studies, the effects of excessive smartphone use can be investigated in the broader age range. In this way, the generalizability of the findings to a wider population can be increased. Additionally, in future studies, the effects of HSU can be evaluated with audiovestibular tests (such as video head impulse test, vestibular evoked myogenic potentials or central auditory tests; temporal resolution and sequencing tests) in addition to psychometric evaluation. Thus, the causes of self-reported auditory and vestibular symptoms can be discussed.

## Conclusion

The study findings demonstrate that individuals with high smartphone use are more likely to experience hearing, tinnitus, balance, falling, and anxiety problems compared to those with low smartphone use. Therefore, high smartphone use can be considered a potential risk factor for individuals experiencing these issues. Further research is needed to understand the underlying mechanisms that lead to these negative effects and to develop effective interventions to mitigate them.

## Data Availability

Data Access Statement: the data collected and presented in the current study are not available publicly or by request due to privacy and ethical concerns.
